# A Review of Food Bioactives That Can Modulate miRNA Profiles for Management of Colorectal Cancer

**DOI:** 10.3390/foods14081352

**Published:** 2025-04-14

**Authors:** Xiaoqin Wan, Tolulope Joshua Ashaolu, Mao-Cheng Sun, Changhui Zhao

**Affiliations:** 1 College of Food Science and Engineering, Jilin University, Changchun 130062, China; 2 Institute for Global Health Innovations, Duy Tan University, Da Nang 550000, Vietnam; tolulopejoshuaashaolu@duytan.edu.vn; 3 Faculty of Medicine, Duy Tan University, Da Nang 550000, Vietnam; 4 College of Food Science and Engineering, Changchun University, Changchun 130022, China

**Keywords:** colorectal cancer, microRNAs, food bioactive components, prevention, treatment

## Abstract

Colorectal cancer (CRC), the third leading cause of cancer globally, with high mortality, necessitates more effective treatments and adjunct therapies. MicroRNAs (miRNAs), short non-coding RNAs, regulate gene expression. Food-derived active components have the potential to modulate CRC cellular processes, aiding in the prevention and management of CRC. This review explores the role of miRNAs in CRC and summarizes the anti-inflammatory, antioxidant, and pro-apoptotic effects of typical food bioactive components by modulating specific miRNAs. We investigate the potential and scientific basis of regulating miRNA expression through dietary therapy and preventive approaches, providing new directions for CRC treatment. Collectively, miRNAs regulate gene expression, impacting the onset, progression, metastasis, and treatment response of CRC. Food components such as curcumin and resveratrol modulate specific miRNAs, affecting CRC cell behavior. Bioactive food components influence CRC cell proliferation, apoptosis, and drug sensitivity by regulating key proteins and pathways.

## 1. Introduction

Colorectal cancer (CRC) is a type of cancer that affects the colon (also known as the large intestine) or rectum. It is estimated that CRC is the third most common cancer worldwide, accounting for approximately 10% of all cancer cases and the second leading cause of cancer-related deaths worldwide [1]. By 2040, the global burden of CRC is projected to increase to 3.2 million new cases per year (an increase of 63%) and 1.6 million deaths per year (an increase of 73%) [1].

In recent years, significant advancements have been made in the research and treatment of colon cancer. However, the survival rate is yet to improve significantly, as the current 5-year survival rate is approximately 60% [2]. The standard treatment strategy for CRC is surgical resection combined with chemoradiotherapy. However, recurrence after curative resection remains a significant concern. Furthermore, the utilization of chemotherapy CRC treatment may result in cytotoxic side effects and the emergence of drug resistance, underscoring the necessity for the development of more efficacious and newer treatments and adjuvants [3].

Incorporation of natural sourced formulations has been associated with effective anti-cancer treatment outcomes with fewer adverse effects [4]. Consequently, dietary natural ingredients play a pivotal role in the fight against cancer. Therefore, it is paramount to explore the effects of natural food bioactive ingredients on CRC due to their potentials.

Meanwhile, a body of evidence indicates that microribonucleic acid (microRNA, μRNA, or miRNA) interactions affect the pathogenesis of CRC [5]. Currently, miRNAs and their target genes are frequently used as biomarkers or therapeutic targets for tumor therapy and have emerged as promising predictors of tumors [6]. Interestingly, many dietary factors have shown promise in manipulating miRNAs associated with CRC. For example, tea polyphenols, a natural polyphenolic phytochemical mainly present in tea leaves, exert anti-inflammatory, antioxidant, and pro-apoptotic effects by regulating specific miRNAs such as miR-93 and miR-328, and have potential preventive and therapeutic effects on CRC [7]. Calycosin, an isoflavone from Radix astragali, affects miRNA in CRC cells, which in turn influences the progression of the cancer. Specifically, calycosin has been shown to downregulate the expression of miR-95 in CRC cells [8]. This is significant because miR-95 is often upregulated in CRC and contributes to tumor growth, invasion, and metastasis. Therefore, the objective of this review is to examine the modulatory impact of diverse food-derived bioactive ingredients or compounds on CRC through the lens of miRNA intervention.

## 2. Methodology

A systematic literature search was conducted to comprehensively identify and evaluate studies related to the modulation of miRNAs by dietary components in the context of CRC management. The search was performed using three major academic databases: PubMed, Web of Science, and Embase, covering the period from 2011 to 2025. The keywords used for the search included “colorectal cancer”, “miRNA”, “food bioactives”, “dietary intervention”, “chemoprevention”, and “miRNA regulation”.

## 3. Colorectal Cancer and miRNAs

### 3.1. Colorectal Cancer

**Figure 1:**
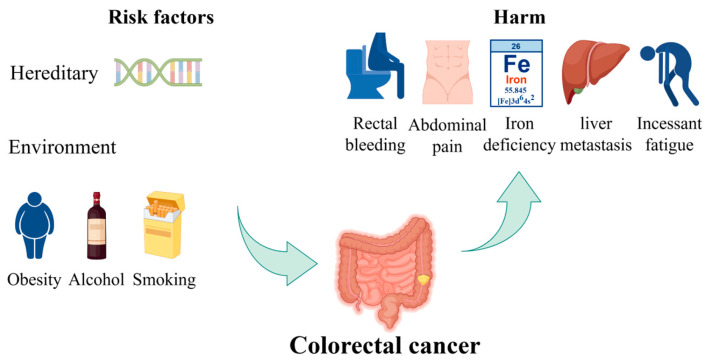
The risk factors of CRC and their harmful impacts. Hereditary factors and environmental influences, such as obesity, alcohol consumption, and smoking, contribute to the risk of developing CRC. The figure also illustrates the potential harms, including rectal bleeding, abdominal pain, iron deficiency, liver metastasis, and persistent fatigue. Part of the elements in the figure are provided by Figdraw.

CRC is one of the most prevalent types of cancer worldwide [9]. The risk of developing CRC increases with age and has the potential to cause significant harm and death. Most CRC cases are diagnosed in individuals over the age of 50 [10]. The incidence and mortality rates are approximately 25% lower in women than in men. These rates also exhibit geographic variation, with the highest rates observed in the most developed countries [11]. There are two principal categories of risk factors of CRC: hereditary and environmental factors. Hereditary factors significantly influence CRC development, with 20–30% of CRCs having a hereditary component. The risk is categorized based on the number and age of onset of affected first-degree relatives (FDRs). High familial risk suggests a genetic predisposition, managed by gene-specific guidelines. Pathogenic germline variants are found in over 7% of CRC cases through next-generation sequencing studies [12]. Environmental factors pertain to individual habits or lifestyles. Some unhealthy lifestyles, including excessive alcohol consumption, a diet high in processed meats and low in fruits and vegetables, sedentary behavior, smoking, and obesity, have been linked to an increased risk of CRC. In its early stages, CRC often lacks symptoms, but certain symptoms may indicate an increased risk, including changes in bowel habits, rectal bleeding, abdominal pain, unexplained weight loss, feeling constantly tired, and iron deficiency anemia [13]. The risk factors of CRC and their harmful impacts are shown in Figure 1. Furthermore, the occurrence of distant metastasis, liver metastasis, and lung metastasis in colon cancer represents a significant cause of mortality, resulting in the failure of colon cancer treatment and ultimately leading to patient death [14].

**Figure 2:**
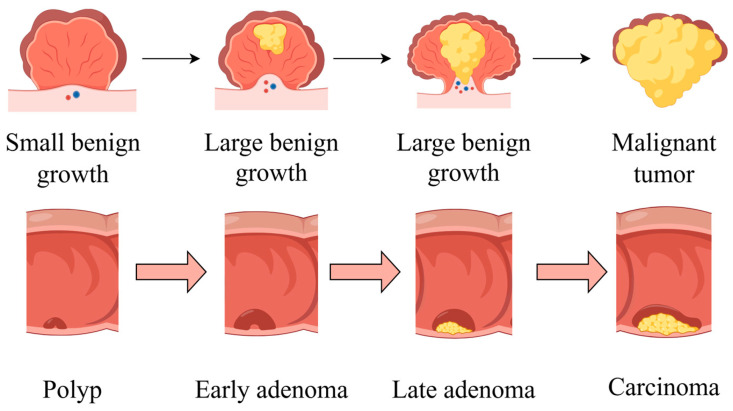
An overview of CRC pathogenesis. The cancer begins with a small benign growth, which is a polyp, then develops into larger benign growths, known as early adenomas. Over time, these can evolve into late adenomas, and eventually, if left untreated, transform into malignant tumors or carcinomas. Part of the elements in the figure are provided by Figdraw.

The development of CRC is characterized by four stages: initiation, promotion, progression, and metastasis. An overview of CRC pathogenesis is shown in Figure 2. The liver is the most common metastatic site, followed by the lung and bone. While the precise duration of each stage remains uncertain, it is probable that decades will be required for the formation of CRC [15].

### 3.2. Effect of miRNAs on Colorectal Cancer

MiRNAs are small clusters of non-coding RNAs with a length of 18–22 nucleotides. They represent one of the most abundant classes of regulatory genes in mammals and exhibit conservation across different species. They are pivotal regulators in the development of living organisms. MiRNAs primarily function by binding to the 3′-untranslated region of a target tumor suppressor gene or oncogene. They play a role in various cellular processes, including proliferation, development, differentiation, senescence, and apoptosis. MiRNAs are both positive and negative regulators of cancer metastasis [16]. In the context of CRC, the regulatory function of miRNAs is multifaceted. They play a crucial role as epigenetic regulators in the initiation, progression, metastasis, and treatment response of cancer by regulating gene expression [17]. The regulatory mechanism of miRNAs on CRC can be categorized based on drug resistance, apoptosis, stability of chromosomes, cell cycle, regulation of the tumor microenvironment, and chemotherapy sensitivity.

#### 3.2.1. Drug Resistance

Drug resistance represents a significant factor contributing to poor prognoses in the treatment and progression of human colon carcinomas. MiRNAs have been increasingly identified as regulators of tumorigenesis and drug resistance. The emergence of drug resistance in CRC is a complex process involving multiple molecular mechanisms, including aberrant expression of miRNAs and alternative messenger RNA (mRNA) splicing events [18]. In particular, the families of miR-200, miR-34a, miR-155, and miR-17 appear to be among the key miRNAs involved in CRC drug resistance. The members of the miR-200 family (miR-200a/b/c and miR-141) and miR-181a play a pivotal role in the multidrug resistance of CRC [19]. These miRNAs have been identified as suppressors of cancer growth and metastasis, exerting their effects through the regulation of various molecular pathways. 5-fluorouracil (5-FU) is a widely used drug for treating CRC patients. Resistance to 5-FU is one of the main reasons for the failure of CRC treatment [20]. By simultaneously delivering 5-FU and miR-21 inhibitor oligonucleotide (miR-21i) to human epidermal growth factor receptor 2 (Her2)-expressing cancer cells, the miR-21-induced cell cycle arrest and tumor proliferation were downregulated while apoptosis increased [20]. Thus, co-delivery of miRNAs with conventional CRC treatment drug can foster an effective therapeutic impact.

#### 3.2.2. Apoptotic Mechanism

Apoptosis is the process of programmed cell death that occurs during normal development and aging. It is a homeostatic mechanism that maintains cell populations by ensuring the destruction of damaged cells. This process can prevent the development of cancerous cells by preventing the survival of damaged cells, thus maintaining cellular homeostasis [21]. In the absence of this process, the damaged cell may evade destruction and instead develop into a cancer cell. Cancer cells are capable of evading apoptosis and undergoing continuous division. A number of miRNAs have been identified as either promoters or inhibitors of apoptosis in CRC. In particular, the families of miR-92a, miR-766, miR-100, miR-21, and miR-32 are reportedly among the key miRNAs involved in regulating the apoptotic process of CRC cells through different mechanisms [22].

#### 3.2.3. Stability of Chromosomes

Chromosomal instability (CIN) is defined as an increased frequency of alterations in chromosome structure or number and is considered a hallmark of cancer. Non-coding RNAs (ncRNAs), including miRNAs and long ncRNAs (lncRNAs), have been identified as key contributors to CIN. The miR-22, miR-26a, miR-28, and miR-186 families are associated with crucial checkpoint proteins involved in maintaining chromosomal stability. CIN contributes to tumor heterogeneity, enabling phenotypic adaptation of cancer cells and promoting therapy resistance and metastasis. A number of miRNAs and lncRNAs that have been demonstrated to play a causal role in CIN may represent promising novel therapeutic targets [23]. For example, METTL16-mediated m6A modification enhances Soga1 expression to maintain chromosomal stability in CRC. This process is critical for accurate chromosome segregation, and its disruption can lead to mitotic abnormalities and CIN [24].

Furthermore, a multitude of potentially beneficial miRNA biomarkers have been demonstrated to be stable in healthy individuals. They possess considerable potential to be regarded as non-invasive biomarkers for diverse cancers, as they can be obtained expeditiously and with minimal risk in biological body fluids, including saliva, serum, and urine [25]. Consequently, miRNAs are promising predictors of tumors.

#### 3.2.4. Cell Cycle Modulation

The impact of the cell cycle on CRC is crucial, as it is directly linked to the proliferation and survival of tumor cells. Key regulatory factors of the cell cycle in CRC, especially Cyclin-Dependent Kinase 4 (CDK4) and Cyclin-Dependent Kinase 6 (CDK6) [26], often lose their normal function due to mutations or overexpression, leading to uncontrolled cell proliferation. MiRNAs can target these types of regulators. For example, miRNAs such as miR-34c-5p and miR-4711-5p can induce cell cycle arrest and apoptosis at the G0/G1 stage by targeting CDK4/6, thereby inhibiting tumor cell proliferation and survival [27,28]. In addition, the combination of these miRNAs with chemotherapeutic agents such as irinotecan and 5-Fu can be a new synergistic strategy for CRC treatment. Therefore, cell cycle-associated miRNAs play a central role in the development of CRC. Also, miRNA mimetics may be used as adjuvant-based therapy, providing a promising research direction to supplement the currently used molecular inhibitors and chemotherapy drugs.

#### 3.2.5. Tumor Microenvironment Regulation

The tumor microenvironment refers to the non-cancerous cells and components present in the tumor, including the molecules they produce and release [29]. The tumor microenvironment is closely related to tumorigenesis because it contains tumor cells that interact with surrounding cells through the circulatory and lymphatic systems, thereby affecting the occurrence and development of cancer. In addition, non-malignant cells in the tumor microenvironment play a critical role in all stages of carcinogenesis by stimulating and promoting uncontrolled cell proliferation [30]. MiRNAs play an important role in regulating key cell types in the tumor microenvironment, such as cancer-associated fibroblasts and immune cells, as well as influencing biological processes such as inflammation and angiogenesis [31].

##### Proliferation

Tumor growth is sustained by cancer stem cells [32]. A continuous proliferation of these cancer cells is a key factor in the development and deterioration of malignant tumors. In CRC, specific miRNAs play an important role in cancer cell proliferation by regulating different molecular pathways. For example, miRNA-148b can positively regulate the proliferation of CRC cells by targeting the CCK2R and p55PIK genes [33]. The CCK2R gene acts as a target of miR-148b, which affects the biological behavior of CRC cells and subsequent regulation. P55PIK is a downstream effector molecule of p53, typically involved in regulating the cell cycle and responding to DNA damage. In addition, miRNA-124 inhibits the cell cycle progression of cancer cells by targeting multiple tumor-associated genes, including CDK6, thereby reducing cell proliferation ability. MiRNA-124 can also enhance oxidative stress and induce cancer cell apoptosis and autophagy, thereby inhibiting tumor growth [34]. The expression of these miRNAs is closely related to the development of CRC, providing new molecular targets for the diagnosis and treatment of CRC.

##### Angiogenesis

Angiogenesis is the process of forming new blood vessels from pre-existing vascular networks and is crucial for various physiological and pathological states, such as embryonic development, tissue repair, and tumor growth [35]. MiRNAs can target angiogenic factors such as vascular endothelial growth factor (VEGF) and fibroblast growth factor (FGF); regulate the functions of endothelial cells, including their proliferation, migration, and differentiation; affect the stability of new blood vessels by targeting molecules involved in vascular maturation; and participate in angiogenic signaling pathways like PI3K-Akt and MAPK/ERK [36]. Also, metastasis, the main cause of death in CRC patients, is regulated by tumor cell angiogenesis [37]. Thus, miRNAs can modulate this process. For example, miR-524-5p inhibits angiogenesis in CRC by targeting WNK1, reducing tumor cell proliferation, inducing cell cycle arrest, and decreasing the production of vascular endothelial growth factor, thereby exhibiting inhibitory effects on tumor growth in both in vitro and in vivo experiments [38].

##### Epithelial–Mesenchymal Transition

Epithelial–mesenchymal transition (EMT) is a critical biological process involving the transformation of epithelial cells into a mesenchymal cell phenotype that plays a vital role in embryonic development, tissue repair, fibrosis, and the invasion and metastasis of cancer. EMT plays a crucial role in tumor invasion and metastasis, aids the detachment of cancer cells from the primary tumor, enters the circulatory system, and forms new tumor foci at a distance [39]. Specific miRNAs act as regulators and affect the expression of genes associated with EMT by inhibiting or promoting the process [40]. For instance, miR-205 can suppress the proliferation, migration, and invasion of CRC cells by targeting MDM4, a negative regulator of p53, and may also inhibit the EMT process by affecting the expression of epithelial markers (like E-cadherin) and mesenchymal markers (like N-cadherin and vimentin) [41]. Therefore, miRNAs act as critical regulators in the control of EMT and may represent potential therapeutic targets for cancer treatment.

#### 3.2.6. Chemotherapy Sensitivity

Chemotherapy sensitivity refers to the responsiveness of tumor cells to chemotherapeutic drugs, that is, the ability of cancer cells to be inhibited or destroyed after exposure to chemotherapeutic drugs. Chemotherapy sensitivity is an important indicator for evaluating the effectiveness of chemotherapy, including effective penetration into tumor tissue, contact with tumor cells, and cancer cell death [42]. In chemotherapy sensitivity, miRNAs can play an important role because they can regulate genes associated with cancer cellular processes such as proliferation, apoptosis, migration, and invasion, thereby affecting the sensitivity of tumor cells to chemotherapy drugs [43]. For example, miR-128-3p reduces the stem cell characteristics and drug efflux ability of tumor cells by downregulating the expression of Bmi1 and MRP5, thereby enhancing the sensitivity of CRC cells to oxaliplatin and making chemotherapeutic drugs more effective in killing cancer cells [44]. In summary, chemotherapy sensitivity is a key determinant of cancer treatment efficacy, which also demonstrates the potential of miRNA as a therapeutic target or biomarker in improving chemotherapy outcomes.

Despite advances, inconsistencies in miRNA profiling across studies and limited validation of causal relationships hinder clinical translation. Additionally, tissue-specific miRNA expression complicates biomarker development [45]. Future research should aim to validate the clinical value of these miRNA biomarkers through larger-scale cohort studies and explore the complex regulatory mechanisms of miRNAs in cancer treatment resistance.

## 4. Potential of Food Bioactive Ingredients in Colorectal Cancer Management

The food environment (FE) is associated with the incidence and mortality of CRC. Increased CRC mortality is associated with animal fat, red meat, alcoholic beverages, high caloric foods, and low FE index [46]. Conversely, a Mediterranean diet, characterized by an abundance of fruits, vegetables, whole grains, and olive oil, along with moderate consumption of fish and wine, is associated with reduced risk of developing CRC [47]. This diet has also become a hot topic in current research. In addition, food bioactive components and ingredients interact with the gut microbiota to significantly impact health. Dietary fibers act as prebiotics, promoting beneficial bacteria and affecting the production of short-chain fatty acids, which have physiological effects [12,24,48,49]. Diet changes can alter the microbiota composition, impacting CRC risk [50]. Therefore, food bioactive ingredients hold significant potential in the prevention of CRC due to their rich content of bioactive compounds such as polyphenols, fiber, and various micronutrients. The consumption of a diet rich in these bioactives, therefore, represents a promising strategy for preventing and managing CRC. Currently, three main models have been used to study the effects of specific food bioactive ingredients on CRC. They include cell, animal, and human models.

### 4.1. Cell Models

Cell models are experiments mainly conducted in vitro, using human CRC cell lines such as HCT116, SW480 Caco-2, etc., to study the effects of specific food components on cancer cell proliferation, apoptosis, cell cycle, and drug resistance. These experiments typically involve adding the sample food ingredients directly to cell culture media and observing their effects on cell behavior. Food ingredients used to study CRC- and miRNA-relevant mechanisms in cell experiments include curcumin, baicalin, kaempferol, deoxyelephantopin, and cordycepin, among others (Table 1). For example, Dou et al. [51] conducted some experiments using curcumin in the CRC cell line SW480 to establish an in vitro cell culture model. SW480 cells were exposed to different concentrations of curcumin to evaluate its effects on cell proliferation and apoptosis. Total RNAs were extracted from the cells before and after treatment, followed by employing q-PCR technology to detect miRNA expression changes associated with curcumin treatment. The study showed that curcumin could suppress CRC by inhibiting cell proliferation instead of promoting cell apoptosis. Notably, curcumin suppressed the Wingless/Integrated (Wnt)/β-catenin pathway and downregulated miR-130a, making miR-130a a potential target of curcumin for CRC treatment. In summary, cell experiments are an invaluable tool in biomedical research, providing a controlled, efficient, and ethical means by which to study the complex interactions between food components and cancer cells at a molecular level.

### 4.2. Animal Models

Animal experiments usually employ nude mice or immunodeficient mouse models, establish tumor models by subcutaneous or intraperitoneal injection of CRC cell lines, and then administer food ingredients orally or by injection to study the effects of food ingredients on tumor growth and metastasis. Similar to cell models, food ingredients and bioactives used to explore relevant mechanisms of CRC and miRNAs in animal experiments include curcumin, baicalin, deoxyelephantopin, Ilex rotunda Thunb (WIR), etc. They also include probiotics such as Clostridium butyricum (Table 1). Chen et al. [52] tested the in vivo colitis-associated cancer (CAC)-preventive effect of the water fraction extracted from the roots of I. rotunda (WIR) and evaluated its miRNA-related mechanism. Male or female C57BL/6 mice aged 12 weeks were injected intraperitoneally with azoxymethane (AOM) and drinking water containing dextran sulfate sodium (DSS). From the fourth week of the experiment, the mice were treated with different doses (12.5 mg/kg and 25.0 mg/kg) of WIR water extract for 5 weeks. Concurrently, changes in body weight and survival rates of the mice were monitored to assess the potential effects of WIR on the CAC model mice. The expression levels of miR-31-5p in the murine colon tissues were detected using qRT-PCR technology to explore the impact of WIR on miRNA expression. Results showed that 25 mg/kg WIR orally administered to mice significantly inhibited atypical hyperplasia and the release and expression of IL-6 and TNF-α in their colon tissues. WIR also impaired in vivo transcription of other pro-inflammatory mediators such as iNOS, IL-11, and IL-17A, whereas same dose of WIR (25 mg/kg) restored and upregulated the miR-31-5p level in the CAC model mice Deductively, animal experiments serve as a crucial bridge in biomedical research, offering an in vivo platform to investigate the intricate effects of food bioactive components on cancer development and progression. Animal models are also essential for translating in vitro findings to a whole-body context, facilitating the development of novel therapeutic strategies and the evaluation of their efficacy and safety before clinical application.

**Table 1:** Food bioactives/ingredients reported to impact colon cancer via miRNA modulation.

Model	Ingredients	Classification	Administration	miRNA	Related Protein or Pathways	Effects on Colon Cancer	References
6-week-old female nude mice were injected with SW480	Curcumin	Curcuminoids	Intraperitoneal injection, 200 mg/kg, once a day for 5 d	miR-130a↓	Wnt/β-catenin↓; Nkd2↑; TCF4↓	Inhibition of CRC cell proliferation	[51]
6-week-old male nude mice (HT-29)	Baicalin	Flavonoids	Intraperitoneal injection, 50 mg/kg and 100 mg/kg, once a day for 21 d	Multiple apoptosis related oncomiRNAs (miR-10a, miR-23a, miR-30, miR-31, miR-151a, miR-205)↓	c-Myc↓; Cleaved Caspase3↑	Inhibition of CRC cell proliferation, induction of apoptosis in tumor cells	[53]
4–6-week-old female BALB/c nude mice were injected with HCT116	Melatonin	Indoleamine neurohormone	Intraperitoneal injection, 25 mg/kg, once a day for 23 d	miR-34a/449a cluster↑	B-cell lymphoma 2 (Bcl-2), Notch1↓; cleaved caspase-3 and cleaved poly (ADP-ribose) polymerase 1 (PARP)↑	Inhibition of CRC cell proliferation and induction of apoptosis in tumor cells	[54]
Human CRC cell lines (HCT8, HCT8-R), human normal colorectal mucosal cell (FHC)	Kaempferol	Flavonoids	Added, IC50 values: 70.39 ± 1.15 μM, 48 h	miR-326↑	PKM2↓; the three splicing factors of the PKM gene (PTBP1, hnRNPA1, and hnRNPA2)↓	Inhibition of colon cancer HCT8-R cells proliferation, induction of apoptosis in tumor cells, overcoming resistance of 5-FU therapy	[55]
25 ± 20 g immunodeficient mice were injected with HCT116	Deoxyelephantopin	Sesquiterpene lactone	Intraperitoneal injection, 30 mg/kg, once every two days for 15 d	miR-205↑	Bcl2↓ change in protein levels associated with cell cycle arrest and apoptosis (CDK1, CyclinB1, C-caspase3, C-PARP)	Promotion of apoptosis in colon cancer cells, a synergistic effect with 5-FU, enhancement in chemosensitivity of colon cancer to 5-FU	[56]
Human CRC cell lines (HCT116, Caco-2)	Cordycepin	Nucleoside analogue	Diluted with dimethyl sulfoxide (DMSO) followed by adding 100 μM for 72 h	miR-26a↑	MYC gene↓	Inhibition of CRC cell proliferation	[57]
Human CRC cell lines (HCT-116, Caco-2)	Quercetin	Flavonoids	The IC50 values: HCT-116: Quercetin = 12.36 µg/mL, 5 FU = 125 µg/mL, Caco-2: Quercetin = 15 µg/mL, 5-FU = 133 µg/mL for 48 h	miR-27a↓	Wnt/β-catenin↓; cyclin D1↓; secreted frizzled-related protein 1 (SFRP1)↑	Promotion of apoptosis in colon cancer cells, a synergistic effect with 5-FU, enhancement in chemosensitivity of colon cancer to 5-FU	[58]
Female BALB/c nude mice were injected with DLD1	Epigallocatechin Gallate (EGCG)	Flavan-3-ols	Subcutaneous injection, EGCG: 25 mg/kg, 5-FU: 20 mg/kg for 14 d	miR-155-5p↑	GRP78↓; nuclear factor-kappa B (NF-κB) ↑; MDR1 ↓; 5-FU accumulation↑	Enhancement in chemosensitivity of colon cancer to 5-FU, promotion of cancer cell apoptosis and DNA damage	[59]
C57BL/6 mice (8 weeks old, weighing 18–22 g), chemical induction	Clostridium butyricum	Clostridium	Oral gavage, 109 CFU of Clostridium butyricum in 500 μL PBS, once a day	miR-200c↑	Proteins involved in EMT (ZEB, p53) and metastasis (HMGB1, VEGFR, ZNF217)↓	Inhibition of CRC cell proliferation, regulation of intestinal barrier function, direct anti-inflammatory actions, inhibition of CRC cell metastasis	[60]
BALB/c female nude mice (5 weeks old) were injected with HCT116	Polyphenols from Hippophae rhamnoides (HPs60)	Flavonoids, phenolic acids	Oral gavage, 50 mg/kg for 21 d	hsa-miR-195-5p, hsa-miR-497-5p↑ hsa-miR-1247-3p↓	cyclinD1, cyclinD2, cyclinD3, cyclinE1, Bcl-2↓; caspase-2↑	Inhibition of tumor growth in vivo, induction of cell cycle arrest and apoptosis	[61]
6- to 8-week-old male nude mice were injected with HT-29 and HCT-116	Calcitriol	Secosteroid	Intraperitoneal injection: 0.1 mg and 0.4 mg; Irinotecan: 50 mg/kg for 2 d	miR-627↑	CYP3A4↓	Enhancement of the efficacy of irinotecan in growth inhibition and apoptosis induction	[62]
Human CRC cell lines (Caco-2)	Coffee (Colombian Arabica) and its constituents (caffeine, caffeic acid, chlorogenic acid, trigonelline)	Phenolic acids	Cell culture exposure, 3.75%, caffeine, caffeic acid, chlorogenic acid, trigonelline: 100 µM each	miR-30c, miR-96↑	KRAS↓	Inhibition of Caco-2 cell proliferation, reduction in KRAS activity	[63]
3-week-old male Wistar rats (chemical induction)	Catalpol	Iridoid glycosides	AOM: intraperitoneal injection, 15 mg/kg, once a week for 14 d; catalpol: intragastric, once a day from week 5 to 25	miR-34a↑	SIRT1↓	Suppression of autophagy and promotion of apoptosis in CRC cells	[64]
Human CRC cell line (HCT-116)	Celastrol	Triterpenoids	Dissolved in DMSO to a final concentration of 50 mM for 72 h	miR-21↓	PCNA, p-Akt, p-GSK3β↓	Inhibition of CRC cell proliferation, induction of apoptosis in tumor cells	[65]
Human CRC cell lines (HepG2, HT-29)	Diterpenoid anthraquinones: Cryptotanshinone (CPT) and Dihydrotanshinone (DHT)	Terpenoid–anthraquinone hybrid compounds	Addition of CPT (5 μM) or DHT (5 μM) in DMEM containing 1% FBS for 72 h	miR-15a-5p, miR-100-5p, miR-200a-5p, miR-210-5p↓	Apoptosis-associated proteins (caspase 3, caspase 7, caspase 9)↑; PARP↓	Inhibition of CRC cell proliferation, induction of apoptosis in tumor cells	[66]
Human CRC cell line (HCT-8)	Ethanol extract of Spica Prunellae (EESP)	Phenolic acids	Added. IC50 values: 0.77 mg/mL for 48 h	miR-34a↑	target genes Notch1, Notch2, and Bcl-2↓	Induction of apoptosis in tumor cells	[67]
Male BALB/c nude mice (6–8 weeks old, weighing approximately 20–24 g), were injected with SW1116	Formononetin	Flavonoids	Intragastric administration, 15 mg/kg, once a day for 14 d	miR-149↑	EphB3, cyclin D1, matrix metalloproteinases 2 and 9↓	Inhibition of CRC cell proliferation, inhibition of the invasive capacity of colon carcinoma cells, cell cycle arrest at the G0-G1 phase	[68]
Human CRC cell line HCT-8 and its multidrug-resistant variant HCT-8/Fu	Bound polyphenol from foxtail millet bran (BPIS)	Phenolic acids	Dissolved in DMSO, IC50 values: 0.77 mg/mL for 24 h	miR-149↑	DNA methyltransferases (DNMT3a, DNMT3b), MECP2, AKT, Cyclin B1, and CDK1↓	Cell cycle arrest at the G0-G1 phase, enhancement in the chemosensitivity of HCT-8/Fu cells to chemotherapeutic drugs	[69]
Human CRC cell lines (SW480, SW620)	Matrine (MAT)	Alkaloids	Added. IC50 values: 1 mM for 24 h	miR-22↑	β-catenin, MEK, ERK↓; cell cycle related proteins CyclinD1, CDK6↓	Inhibition of CRC cell proliferation, induction of apoptosis in tumor cells, cell cycle arrest at the G0-G1 phase	[70]
8-week-old Balb/c nude mice were injected with 5-FU-resistant DLD-1 cells	5-FU, erlotinib	Drug	Intraperitoneal injection, 5-FU, erlotinib: 40 mg/kg each, twice a week, 5-FU: 9 weeks, erlotinib: 10 weeks	miR-330-3p↑	EGFR Protein, FGD5-AS1, HK2 Protein↓	Inhibition of CRC cell proliferation, enhancement in chemosensitivity of colon cancer to 5-FU, regulation of the tumor microenvironment	[71]
4-week-old female BALB/c nude mice were injected with SW620 and HCT-116	Ginsenoside Rh2	Triterpenoid saponins	Intraperitoneal administration, 50 nM every other day for 42 d	miR-150-3p↑	The pro-apoptotic genes Bax and cleaved caspase-3↑; PCNA, survivin, cyclin D1, Myc, β-catenin and SRCIN1↓	Inhibition of colon cancer cell proliferation, migration, invasion, and induction of apoptosis in tumor cells	[72]
Human CRC cell lines (COLO205 and SW480)	Estradiol	Steroid hormone	Added in different concentrations for 48 h	miR-31, miR-155, miR-135b↓	ER-β, hMLH1, hMSH2, apoptotic cells (Annexin-V+/PI-)↑	Inhibition of the colon cancer cell proliferation, invasion, and induction of apoptosis in tumor cells	[73]
Human CRC cell line (Caco-2)	Exopolysaccharides (EPSs)	Polysaccharides	Added in different concentrations (200 μg/mL, 400 μg/mL, 600 μg/mL, 800 μg/mL, and 1000 μg/mL) for 48 h	miR-155↓	TP53, AIFM1, Rb1↑; Bcl2, KI-67, c-myc, K-Ras, β-catenin, mTOR, LC3A↓	Inhibition of CRC cell proliferation, induction of apoptosis in tumor cells, cell cycle arrest at the G1-S phase	[74]
Human CRC cell line (HCT-116)	Butyrate	Organic acids	Added, 1–2 mM for 24–48 h	miR-106b family↓	p21 protein↑	Inhibition of CRC cell proliferation	[75]
6-week-old C57BL/6J mice (chemical induction)	Cucurbitacin E (CE)	Triterpenoids	Oral gavage,1 mg/kg, once a day for 7 d	miR-371b-5p↑	Impact on cell cycle proteins; senescence-associated proteins such as p53, p27, and p21↑; TFAP4↓	Inhibition of CRC cell proliferation and induction of cell senescence	[76]
Human CRC cell line (DLD-1)	Three main propolis cinnamic acid derivatives: Artepilin C, Baccharin, and Drupanin	Phenolic acids	Added and dissolved in DMSO for 48 h	miR-143↑	Activation of apoptosis-related proteins and apoptotic signaling pathways; impact on cell cycle and cell survival-related proteins; Erk5 and c-Myc↓	Inhibition of CRC cell proliferation, induction of apoptosis in tumor cells, synergistic effect of Baccharin and Drupanin	[77]
6-week-old male C57BL/6J mice (chemical induction)	Avenanthramide A (AVN A)	Phenolic amides	Oral gavage, 30 mg/kg, once a day for 7 d	miR-129-3p↑	Regulation of cell cycle proteins; p53 protein levels, p21 protein↑; Pirh2 protein, IGF2BP3, and CDK6↓	Inhibition of CRC cell proliferation, induction of cell senescence, cell cycle arrest	[78]
Human CRC cell lines (HCT116, LOVO, and DLD-1)	A hexane extract of American ginseng (HAG)	Organic acids	Added at 260 mg/mL for 24 h	miR-29b↑	MMP-2↓	Inhibition of CRC cell proliferation and migration, induction of cell senescence	[79]
Human colon-derived CCD-18Co myofibroblast cells	A polyphenolic extract (WE) from red wine made with Lenoir grapes (Vitis aestivalis hybrid)	Proanthocyanidins, anthocyanins	Added. WE was diluted to 25–100 mg/mL for 24 h–72 h	miR-126↑	NF-kB Protein↓; cell adhesion molecules ICAM-1, VCAM-1, and PECAM-1 induced by LPS↓; VCAM-1 protein↓	Anti-inflammatory effects, antioxidant effects	[80]
5-week-old male Balb/c nude mice were injected with HCT 116	Metformin	Drug	Oral gavage at 25 mg/kg, once a day for 14 d	miR-361-5p↑	MYC↓; Sonic Hedgehog signaling pathway↓	Inhibition of CRC cell proliferation, enhancement in chemosensitivity of colon cancer to 5-FU and oxaliplatin, reduction in stem cell marker expression	[81]

Notes: ↑ upregulated; ↓ downregulated; CRC = colorectal cancer; EMT = epithelial–mesenchymal transition.

### 4.3. Human Models

Human experiments typically involve small-scale clinical studies investigating the effects of food ingredients on CRC patients. These experiments need to strictly adhere to ethical guidelines and be conducted with the patient’s consent. For example, in a recent study, individuals with familial adenomatous polyposis (FAP) who had mutations in the APC gene were chosen as participants. The participants underwent a three-month dietary intervention that adhered to the principles of the traditional Mediterranean diet, with an emphasis on reducing pro-inflammatory foods and incorporating more fermented foods, inspired by Japanese culinary traditions. Throughout the study, participants provided fecal and blood samples at three different time points: before the intervention began (T0), three months after the intervention started (T1), and six months after the intervention concluded (T2). Small RNA sequencing was utilized to evaluate the alterations in miRNA expression in the fecal samples collected at these intervals [82]. It was observed that the diet modulated 29 fecal miRNAs persisting for three months after the intervention. MiR-3612-3p and miR-941 levels matched diet adherence; miR-3670 and miR-4252-5p corresponded with fecal calprotectin; and miR-3670 and miR-6867 were in agreement with serum calprotectin. Seventy different genes were expressed between adenoma and normal tissue before the dietary intervention but reached similar levels after the experiment. Pro-inflammatory ERK1/2, cell cycle regulation, and nutrient response pathways were observed based on functional enrichment analysis as the regulatory mechanism of miRNAs and the genes. The study suggests that changes in miRNAs levels and genes with oncogenic and tumor suppressor functions support cancer-preventive effect of Mediterranean diet. Usually, the data from human experiments is crucial for verifying the results of cell and animal experiments, as they provide direct evidence about the effects of food components in the human body. However, due to ethical and practical limitations, the number of human experiments is usually limited and often focused on evaluating the safety and tolerability of food ingredients.

### 4.4. Methodological Considerations and Limitations

Cell models, while invaluable for initial screenings and mechanism exploration, have notable limitations. For instance, the doses of bioactive compounds used in vitro far exceed physiological levels from human dietary intake, potentially masking true effects and interactions at normal doses. Moreover, these models often oversimplify the tumor microenvironment by lacking the complex interplay of immune cells, stromal cells, and extracellular matrix components, which are crucial for accurately mimicking in vivo conditions and understanding tumor behavior and drug responses [83]. When using animal models, species differences, such as the immune responses of immunodeficient mice vs. humans, affect treatment efficacy and safety. For example, nude and Severe Combined Immunodeficiency (SCID) mice, used in xenografts, lack a functional immune system, limiting the translation of preclinical findings [84]. Dose translation is also challenging; mouse carcinogen doses (AOM, DSS) do not directly apply to humans due to differences in metabolism, body weight, and surface area, complicating human trial dosing based on animal studies [85]. In current research, the sample size of human experiments is relatively small. The small sample size may lead to randomness and bias in the results, making it difficult to reflect the true situation of the entire population. In addition, the intervention period is relatively short, which limits the evaluation of long-term effects. Short term interventions may not fully observe the potential benefits of chronic disease prevention or treatment, thereby affecting the generalizability and clinical application value of the results [86]. Therefore, future research needs to further optimize methodological design to overcome these limitations and provide more reliable basis for research and applications in related fields.

## 5. Proposed Mechanisms

Based on the aforementioned studies on the food ingredients employed in CRC experiments, several mechanisms were frequently observed, including the regulation of Wnt/β-catenin signaling pathway, NF-κB signaling pathway, cell cycle-related proteins, apoptosis-related proteins, EMT-related proteins, angiogenesis-related factors, multidrug resistance-related proteins, cell proliferation and survival-related proteins, and cell senescence-related proteins.

### 5.1. Wnt/β-Catenin Signaling Pathway

The Wnt/β-catenin signaling pathway is a family of proteins that play a crucial role in embryonic development and adult tissue homeostasis, and its abnormal activation often leads to a variety of severe diseases, including cancer [87]. In CRC, aberrant activation of this pathway is often due to the loss or inactivation of the Adenomatous polyposis coli gene, leading to the accumulation of β-catenin protein in the cytoplasm and its translocation to the nucleus, where it binds to TCF/LEF family transcription factors, activating downstream Wnt target genes, thereby promoting cell proliferation, survival, and migration, ultimately leading to tumor formation and progression [88]. In this review, the food bioactive ingredients that regulate the Wnt/β-catenin signaling pathway are mainly curcumin and quercetin.

### 5.2. NF-κB Signaling Pathway

NF-κB is a principal inducible protein and a predominant transcription factor that plays a crucial role in controlling gene expression in mammals, especially under certain physiological and pathological conditions [89]. In CRC, the abnormal activation of the NF-κB signaling pathway is closely associated with tumor initiation, progression, invasiveness, and therapeutic resistance. When intestinal epithelial cells are damaged or stimulated by inflammation, the NF-κB pathway is activated, promoting the expression of inflammatory cytokines that can enhance the proliferation, survival, and angiogenesis of tumor cells, thereby playing a role in the progression of CRC [90]. Additionally, NF-κB is involved in mechanisms of resistance to chemotherapy and radiotherapy, complicating the treatment of CRC [91]. In this review, the food bioactive that regulates the NF-κB signaling pathway is EGCG.

### 5.3. p53

p53 is a crucial tumor suppressor protein that regulates a variety of cellular responses to protect against cancer development, including DNA repair, cell cycle arrest, cellular senescence, cell death, cell differentiation, and metabolism [92]. Upon DNA damage, wild-type p53 acts to restrain the process of cell replication until the damage is repaired, thus preventing the propagation of DNA-defective cells and the acquisition of a cancer phenotype. However, when the p53 protein is mutated, the cell cycle becomes unrestricted, and damaged DNA is replicated, leading to uncontrolled cell proliferation and the formation of cancer tumors [93]. The p53 protein also suppresses tumor growth and progression by activating its downstream target genes, such as p21, which promotes cell cycle arrest, and Bax, which promotes apoptosis [94]. In this review, the food bioactives that regulate p53 mainly include Clostridium butyricum, EPSs, CE, and AVN A (from oats).

### 5.4. Bcl-2

Bcl-2 is a family of proteins that play a crucial role in regulating apoptosis, or programmed cell death [95]. In CRC, the de-regulation of Bcl-2 family members is associated with tumor initiation, progression, and resistance to therapy. Members of the Bcl-2 family control the permeability of the mitochondrial membrane, determining whether a cell undergoes apoptosis, and include pro-survival members (such as Bcl-2, Bcl-xL, and Mcl-1) and pro-apoptotic members (such as Bax and Bak) [91]. Moreover, the dysfunction of the BCL-2 family is involved in its interaction with various signaling pathways, including its impact on cell cycle, metabolism, and DNA repair processes, thereby playing a crucial role in the occurrence, development, and treatment of CRC [95]. In this review, the food bioactives that regulate Bcl-2 mainly include HPs60, EESP, and melatonin.

Current research suggests that some dietary bioactive ingredients can affect the CRC process by directly regulating miRNA expression. For example, curcumin significantly downregulates miR-130a by inhibiting the Wnt/β-catenin signaling pathway, thereby inhibiting tumor cell proliferation [51]. However, some studies only observed a correlation, such as coffee components reducing KRAS expression, but did not clarify the miRNA-mediated regulatory mechanism [63]. To clarify the causal relationship, it is necessary to combine functional experiments (such as miRNA overexpression/knockout models, dual luciferase reporter gene detection) in the future to verify the necessity of specific miRNAs in the action of bioactive components, in order to avoid interference from confounding factors and provide reliable evidence for clinical translation.

**Figure 3:**
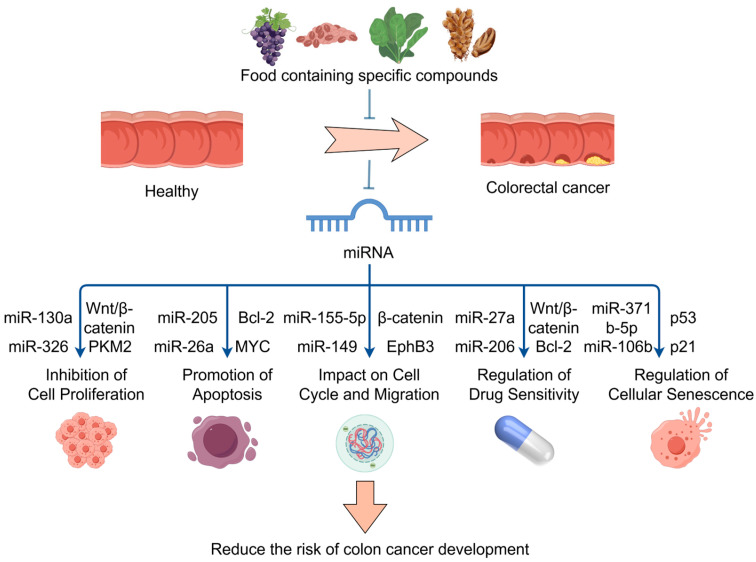
The mechanism by which specific compounds in the diet reduce cancer risk through miRNA regulation; food bioactive ingredients regulate miRNAs. These miRNAs target key genes and pathways, such as the Wnt/β-catenin, to inhibit cell proliferation, promote apoptosis, impact cell cycle and migration, regulate drug sensitivity, and regulate cellular senescence. Part of the elements in the figure are provided by Figdraw.

Therefore, these food bioactive ingredients regulate the expression of specific miRNAs, which are involved in a number of biological processes, including cell proliferation, apoptosis, cell cycle control, drug resistance, EMT, and metastasis. These compounds play a significant role in the onset, progression, and response to treatment of CRC. The mechanism by which specific compounds in the diet reduce cancer risk through miRNA regulation is shown in Figure 3. It is safe to connote that these bioactives and nutritional components of food have high potential use as therapeutic and preventive dietary strategies for CRC by modulating the expression of various miRNAs.

## 6. Conclusions and Future Perspectives

This review highlights the role of active dietary components in the prevention and treatment of CRC with an emphasis on miRNAs as regulators of the disease. The bioactives can modulate the expression of specific miRNAs related to CRC, offering potential for both prevention and treatment. Recent meta-analyses have included 42 observational studies to explore the association between wine consumption and cancer risk. Results showed no significant difference in overall cancer risk between red (RR = 0.98) and white wine (RR = 1.00). However, subgroup analyses revealed that white wine consumption was associated with increased cancer risk in cohort studies (RR = 1.12) and among women (RR = 1.26). For CRC, the analysis showed that there was no significant difference between red and white wine consumption in relation to cancer risk [96]. This suggests that the type of wine consumed may not have a differing impact on CRC risk. However, it is important to note that other factors such as the amount of alcohol consumed, duration of consumption, and individual susceptibility can influence cancer risk. Further research is needed to better understand the complex relationship between alcohol consumption and cancer risk. miRNAs play a complex role in the development of CRC and can serve as therapeutic biomarkers or targets. They primarily function by binding to the 3′-untranslated region of target tumor suppressor genes or oncogenes and are involved in processes such as cell proliferation, development, differentiation, senescence, and apoptosis. In CRC, the regulatory function of miRNAs is multifaceted, including the regulation of drug resistance, apoptosis, chromosome stability, cell cycle, and the tumor microenvironment. Food bioactives such as curcumin, resveratrol, and quercetin show high potential in CRC management by affecting miRNA expression, which in turn influences cancer cell behavior.

These findings provide a scientific basis for the development of new therapeutic strategies and preventive measures for CRC and offer new perspectives on the management of CRC through dietary intervention. Future research needs to further explore the specific mechanisms of interaction between dietary components, miRNAs, and CRC to better utilize this knowledge to improve treatment outcomes and quality of life for patients with CRC.

CRC currently primarily relies on treatments such as surgery, chemotherapy, and radiotherapy, which can be used in combination to enhance therapeutic outcomes. However, these approaches often come with many side effects due to their non-specificity and cytotoxicity towards cells that are growing and dividing. Moreover, a significant proportion of patients still experience relapse even after a series of treatments. Therefore, the development of more alternative and effective treatment methods is crucial for patients with CRC. Immunotherapy, as one of the emerging treatment options, leverages the patient’s own immune system to combat cancer cells, overcoming the specificity issues associated with chemotherapy and radiotherapy, and having a lesser impact on normal cells, offering new hope for the treatment of CRC [97]. The combination of immunotherapy and natural ingredients is an emerging and interesting research field. Some natural ingredients, such as antioxidants and Omega-3 fatty acids, have been found to enhance immune system function and theoretically can be combined with immunotherapy to improve treatment efficacy. In addition, the low toxicity of natural ingredients makes them advantageous and have great potential as adjunctive therapies. Therefore, more research should be directed towards the modulation of miRNA profiles as a dietary strategy for CRC prevention.

Despite the potential of dietary bioactive ingredients in regulating miRNA, their clinical translation still faces multiple challenges. For example, clinical studies showed that curcumin is safe for humans even at high doses, but unfortunately its bioavailability is very low. This makes therapeutic use very limited [98], so it is necessary to optimize absorption through nanoparticle or liposome delivery systems [99]. Another notable example is resveratrol, where only the cis isomer exhibits bioactivity, while the more abundant trans isomer and their glycosidic derivatives (cis-piceid and trans-piceid) lack efficacy [100]. In addition, there is a significant difference between the experimental dose and the actual human intake, and dose standardization needs to be achieved through pharmacokinetic studies. Personalized nutrition strategies can enhance targeting by integrating patient miRNA profiles and genomic features to customize intervention plans. In the future, priority should be given to advancing clinical trials, exploring the synergistic effects of bioactive ingredients and immunotherapy, and utilizing multi-omics techniques to analyze miRNA metabolite interaction networks, in order to promote the application of precision medicine in CRC management.

## Data Availability

No new data were created or analyzed in this study. Data sharing is not applicable to this article.
